# Mr. Long and Vaccination

**Published:** 1903-01-24

**Authors:** 


### The Hospital.

A Journal of

The Medical Sciences and Hospital Administration.

Vol. XXXIII.—No. 852. Edited by Sir Henry Burdett, K.C.B., and Solomon C. Smith, M.D., M.R.C.P. [January 24, 1903.

### Mr. Long and Vaccination.

The reception by the President of the Local Government Board, on the 14th instant, of the deputation which waited upon him on the part of the recently formed Imperial Vaccination League afforded what was probably a unique example of absolute accord between a minister and those making representations to him. Mr. Long declared that his only wonder was that it should be necessary, at this period of the world's history, for a great deputation to come to him in order to advocate opinions which he believed ought to command the unqualified assent of every intelligent person. He hastened, of course, with the instincts of a gentleman, to declare that he meant no offence to those who believed that vaccination was wrong, but continued by saying that the more he studied the question the more difficult he found it to understand how anyone could fail to give a hearty support to one of the greatest triumphs of preventive medicine.

The chief recommendations which the Imperial Vaccination League desired to press upon the attention of the minister, and to see carried out under the sanction of the law, were the removal of the control of vaccination from the hands of the Guardians of the Poor, the compulsory vaccination (save always for the conscientious objector) not only of infants, but also of all children at some convenient time before puberty, say at the age of twelve or thirteen; an official definition of effectual vaccination; and Government control of the supply of glycerinated lymph. It was pointed out that revaccination might with great propriety coincide with the close of school life, and precede the entrance upon any form of industry, so that it might not stand in the way of doing anything necessary to be done. To a law enacting compulsory revaccination, the speakers on the subject did not anticipate any kind of popular resistance. If a child had actually been vaccinated in infancy, and no harm had followed from the operation, parents would probably care little about its being done again. The obstacle to be overcome would not be opposition but neglect, not hostility but indifference. The natural corollary of a law requiring revaccination would be a refusal to allow young persons who had not been revaccinated to enter any department of the public service; and an example thus set would undoubtedly soon be followed by the great majority of private employers. It should, however, with regard alike to vaccination and revaccination, be made part of any national system that the conditions of supposed efficiency should be defined by authority. It is notorious that large numbers of the children now said to be vaccinated are insufficiently protected, in consequence either of too small a number of insertions or of some irregularity in the course of the pustules, and that the chief effect of the application to them of the word is to introduce an element of error into statistics.

On all the points submitted to him by the deputation, Mr. Long, while plainly stating his assent as a matter of personal opinion, was careful to say that he was speaking without consultation with his colleagues in the ministry, and that he was unable to make any announcement with regard to the intentions of the Government. Notwithstanding this, and especially as the approaching expiration of the Act now in force renders it necessary that something should be done, it is reasonable to hope that Mr. Long's personal convictions will be permitted to bring about their natural results. The courage, the indifference to the clamour of old women of both sexes, and the firm reliance upon scientific knowledge, which enabled him to banish rabies and hydrophobia from the country, will be fresh in the recollection of the public; and it may fairly be expected that these qualities will be again displayed in relation to small-pox, and that they will again be productive of their natural or inevitable results. It is the more to be regretted that, upon one point of great importance, the preparation and supply of glycerinated lymph, Mr. Long seemed to be more conscious of the difficulties than of the advantages. The former, whatever they are, should be overcome. Large numbers of people, who are quite able and willing to pay for the vaccination of their children, are now induced, or practically compelled, to take them to the public vaccinator, to be operated upon at the public expense, because they know that their ordinary medical attendant is forced to go into the market for his lymph, and cannot obtain any satisfactory guarantee with regard either to the efficacy of that which is supplied to him, or to the care employed in its preparation. Some months ago, when a complaint was made, during the height of the recent epidemic, that the Government lymph was supplied to public vaccinators alone, and refused to private practitioners, the *Times* described the arrangement as one which bore a greater resemblance to comic opera than to rational administration. The description was an entirely just one; and, now that the epidemic has passed away, we are entitled to hope that for the future, and notwithstanding "difficulties," the principles of rational administration will be permitted to prevail.

